# Antifungal Chemical Compounds Identified Using a C. elegans Pathogenicity Assay

**DOI:** 10.1371/journal.ppat.0030018

**Published:** 2007-02-02

**Authors:** Julia Breger, Beth Burgwyn Fuchs, George Aperis, Terence I Moy, Frederick M Ausubel, Eleftherios Mylonakis

**Affiliations:** 1 Division of Infectious Diseases, Massachusetts General Hospital, Boston, Massachusetts, United States of America; 2 Department of Molecular Biology, Massachusetts General Hospital, Harvard Medical School, Boston, Massachusetts, United States of America; 3 Department of Genetics, Harvard Medical School, Boston, Massachusetts, United States of America; Johns Hopkins, United States of America

## Abstract

There is an urgent need for the development of new antifungal agents. A facile in vivo model that evaluates libraries of chemical compounds could solve some of the main obstacles in current antifungal discovery. We show that *Candida albicans,* as well as other *Candida* species, are ingested by Caenorhabditis elegans and establish a persistent lethal infection in the C. elegans intestinal track. Importantly, key components of *Candida* pathogenesis in mammals, such as filament formation, are also involved in nematode killing. We devised a *Candida*-mediated C. elegans assay that allows high-throughput in vivo screening of chemical libraries for antifungal activities, while synchronously screening against toxic compounds. The assay is performed in liquid media using standard 96-well plate technology and allows the study of C. albicans in non-planktonic form. A screen of 1,266 compounds with known pharmaceutical activities identified 15 (∼1.2%) that prolonged survival of C. albicans-infected nematodes and inhibited in vivo filamentation of *C. albicans.* Two compounds identified in the screen, caffeic acid phenethyl ester, a major active component of honeybee propolis, and the fluoroquinolone agent enoxacin exhibited antifungal activity in a murine model of candidiasis. The whole-animal C. elegans assay may help to study the molecular basis of C. albicans pathogenesis and identify antifungal compounds that most likely would not be identified by in vitro screens that target fungal growth. Compounds identified in the screen that affect the virulence of *Candida* in vivo can potentially be used as “probe compounds” and may have antifungal activity against other fungi.

## Author Summary

Candida spp. are among the most significant causes of nosocomial infections, and disseminated candidiasis continues to have an attributable mortality rate of over 25%. For this reason, we have developed a liquid media assay using the model nematode Caenorhabditis elegans as a model organism for *Candida* infection. The worms are infected on solid media lawns and then moved to pathogen-free liquid media. Unless antifungal compounds are added to the wells, the majority of worms die within 3–4 d. This model is similar to the infection process in humans, in that *Candida* cells are able to produce filaments, which are essential for the infection process in humans. We used this pathogen model to create a semi-automated, high-throughput screen using C. elegans to evaluate the antifungal effectiveness of many types of chemical compounds. Through this process, we have identified three compounds that we show have varying degrees of antifungal activity in *C. elegans,* in vitro, and in mice.

## Introduction

The discovery of substantial commonality between microbial pathogenesis in mammals and nonvertebrate model hosts, such as the nematode *Caenorhabditis elegans,* has provided the foundation for the development of high-throughput genetic analysis of microbial virulence factors in live animal models (reviewed in [1,2]). We sought to extend the use of the C. elegans infection models to identify chemical compounds with antifungal activity against *Candida* species, the most common human pathogenic fungus.

Candida spp. are the fourth most common cause of nosocomial blood-stream infections, and disseminated candidiasis continues to have an attributable mortality rate of over 25% [3]. The mortality rate for device-associated *Candida* infection can be even higher [4]. In the United States, the overall excess cost attributable to candidemia is estimated at $1 billion per year and the average cost of candidemia for a single episode is about $40,000 (1997 United States dollar) [5,6]. However, efforts to identify new antifungal compounds have been hindered by the fact that most compounds that have antifungal activity in vitro are also toxic to mammalian cells. A facile bioassay compatible with high-throughput screening technologies, which simultaneously evaluated libraries of chemical compounds for antifungal activity and host toxicity in the context of a whole-animal *Candida* infection model, could solve some of the main obstacles in current antifungal discovery.

Here, we show that *Candida albicans,* as well as other *Candida* strains, can kill *C. elegans.* We used these findings to develop a C. elegans-based infection model that is performed in standard 96-well plates in liquid media, thereby enabling automated addition of compounds. An important feature of the assay is that the first assay endpoint is nematode survival, which automatically eliminates highly toxic compounds that affect nematode viability. Moreover, important components of *Candida* pathogenesis in mammals are involved in nematode killing, and filamentation becomes apparent as the nematode dies, providing a second clinically relevant endpoint*.* We demonstrate how this model enables the screening of a chemical library for compounds that prolong nematode survival or inhibit *Candida* filamentation. Importantly, the screen identifies compounds that prolong survival in a hematogenous murine model of candidiasis.

## Results

### Killing of C. elegans by *Candida* Species

**Figure 1:**
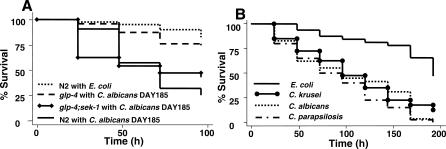
Killing of C. elegans after Exposure to *Candida* Species (A) N2, *glp-4, or glp-4;sek-1* L4 nematodes feeding on E. coli OP50 were transferred to C. albicans strain DAY185 for 2 h and then transferred to pathogen-free liquid medium. Dead nematodes were counted and removed daily. N2 worms transferred from E. coli OP50 directly to liquid media were used as control. Survival of *glp-4;sek-1* is significantly shorter compared to the strain *glp-4* (*p* < 0.001). (B) Survival of *C. elegans glp-4;sek-1* feeding on lawns of C. albicans ATCC#90028, C. parapsilosis ATCC#20019, or C. krusei ATCC#6258. *p* < 0.001 for each of the yeast strains compared to control nematodes that were exposed to E. coli OP50 (∼65 nematodes/group).

C. elegans nematodes eat microorganisms, but they die when fed a variety of human bacterial and fungal pathogens [1,2]. In the laboratory, C. elegans-killing assays are typically carried out by transferring nematodes from lawns of Escherichia coli strain OP50 (their normal laboratory food) to lawns of pathogenic bacteria or yeast grown on solid agar media. However, screening chemical libraries using an agar-based *C. elegans-*killing assay is not readily compatible with the use of robots for filling assay plates and pin transfer of compounds, homogeneous distribution of chemicals in the medium, or the use of automated plate readers or automated screening microscopes to monitor nematode survival. We therefore sought to develop a C. elegans–Candida spp.-killing assay that could be performed in liquid media. We found that when wild-type L4 stage N2 nematodes were fed Candida spp. on solid brain–heart infusion (BHI) medium for 2 h and then were transferred to a liquid medium consisting of 20% BHI and 80% M9 minimal medium, the longevity of the worms was significantly reduced compared to worms not infected with *Candida* (Figure 1A). We tested different times of exposure to Candida spp. on solid BHI medium (from 5 min to 24 h) and in all cases the longevity of the worms was significantly reduced compared to uninfected worms (unpublished data**)**.

Although Candida spp. killed wild-type C. elegans in liquid medium, the killing did not occur rapidly enough to prevent the production of progeny produced by 3- and 4-d-old hermaphroditic nematodes. The presence of a brood in the assay mix made it difficult to score the viability of the infected parents. Indeed, in the assay described above and shown in Figure 1A, it was necessary to painstakingly remove progeny nematodes that survived beyond the L1 or early L2 developmental stage. Production of progeny during the assay had another undesirable consequence in the scoring of the assay; namely, *Candida*-infected wild-type nematodes exhibited significant matricidal death (over 30%) involving the premature hatching of eggs in the C. elegans uterus (which is not necessarily directly associated with an infectious-like process) around day 3 of the experiment. To avoid these problems associated with progeny production, we substituted *C. elegans glp-4* mutants for wild-type in the Candida spp. killing assay. *C. elegans glp-4* mutant animals have normal morphology and brood sizes at 15 °C, but do not make gonads and are unable to produce eggs at 25 °C [7–10]. As shown in Figure 1A, Candida spp. also killed *glp-4* nematodes, but the rate of killing was significantly slower than for wild-type worms (Figure 1A), similar to results reported previously for the killing of *glp-4* and other sterile mutants by bacterial pathogens.

Although *glp-4* mutant worms could have potentially been used in an antifungal screening assay, we sought to increase the rate of killing of the *glp-4* mutant by utilizing *glp-4;sek-1* double mutant worms. *SEK-1* encodes a mitogen-activated protein kinase kinase that functions directly upstream of the C. elegans homolog of the mammalian p38 mitogen-activated protein kinase that was shown previously to be an important component of the C. elegans defense response to pathogens [7,11]. As shown in Figure 1A, the *C. elegans glp-4;sek-1* double mutant was significantly more susceptible to *Candida* than the *glp-4* mutant (*p* < 0.001), suggesting that the C. elegans homolog of the mammalian p38 mitogen-activated protein kinase (which is involved in mammalian response to candidiasis [12]) plays a significant role in the C. elegans response to *Candida.* This finding is consistent with the observation that *sek-1* mutants are more susceptible to a variety of pathogens [7,11]. When *glp-4* and *glp-4l;sek-1* mutants were moved from OP50 plates to pathogen-free liquid media, their survival was not significantly different from N2 worms in the presence of E. coli in liquid media (unpublished data). Notably, when we exposed *glp-4;sek-1* worms to DAY185 for 2 h and then moved to lawns of an E. coli OP50 strain that expresses green fluorescent protein (GFP), over 70% of the worms had no GFP-expressing bacteria in their intestine, suggesting that the grinder is still operational, at least in the majority of the nematodes. Because the *glp-4;sek-1* mutant was similarly susceptible to a variety of C. albicans strains as well as to the non-*albicans* strains Candida krusei and Candida parapsilosis (Figure 1B), because it did not produce progeny, and because we found it compelling to study *Candida* pathogenesis in immunocompromised nematodes (in some ways analogous to candidiasis in immunocompromised humans), we utilized the *glp-4;sek-1* strain in all of the further studies described below.

### Progression of *Candida* Filamentation within C. elegans

*Candida* yeast cells have the ability to develop filaments that are usually differentiated to hyphae (long continuous germ tubes separated by true septin rings) or pseudohyphae (chains of distinct cells that fail to separate). Development of human candidiasis involves adhesion to the substratum (which allows the yeast to colonize surfaces, such as the gastrointestinal tract and intravascular catheters), development of biofilm, and formation of hyphae and pseudohyphae [13–15]. Filamentation is instrumental in *Candida* virulence in mammals; nonfilamentous mutants of C. albicans are highly attenuated [16]. *Candida* hyphae invade host tissues and exert mechanical force through cells and tissues, enabling escape of C. albicans from the host bloodstream [17,18]. Notably, there is significant coregulation between adherence, biofilm, and filament pathways [15,19,20]. For example, genes involved in biofilm formation are required for hyphal development, while filaments are a prominent feature of C. albicans biofilms that support biofilm integrity [4,21–23].

**Figure 2:**
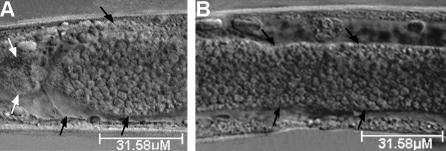
Intact *C. albicans* Cells Persist within the C. elegans Intestine C. elegans strain *glp-4;sek-1* L4 worms were transferred from OP50 to C. albicans for 30 min and then moved to *Candida*-free liquid media for 6 d when the worms were photographed. Intact yeast cells are seen within the (A) proximal and (B) distal intestine. White arrows in (A) point to the pharyngeal grinder organ. Black arrows point to the intestinal lumen. Scale bars are 31.58 μm.

Persistence in the intestine appears to be a critical first step during *Candida* infection in *C. elegans.* Indeed, nematodes exposed to *Candida* lawns are unable to clear the *Candida* infection when they are transferred to *Candida*-free liquid media (Figures 2 and S1). Even when nematodes were exposed to *Candida* lawns for as little as 5 min and then transferred to pathogen-free liquid media or lawns of E. coli OP50, 100% of the nematodes remain colonized (unpublished data). As a result, *Candida* cells fill the nematode pharynx and intestine (Figure S1B and unpublished data). Also, we placed the worms on lawns of C. albicans strain MLR62, a strain that constitutively expresses GFP, and then we moved them onto the non-GFP parent strain DAY185. Over a 24-h period, the GFP-expressing *Candida* cells were displaced by non-GFP-expressing cells, suggesting that *Candida* cells are not too big to be defecated.

**Figure 3:**
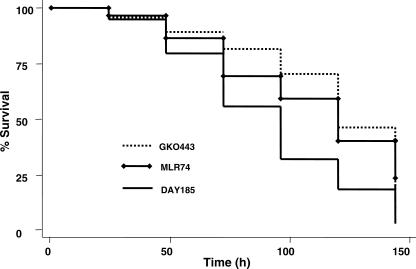
*Candida* Biofilm Formation and Filamentation Is Associated with C. elegans Killing Survival of *C. elegans glp-4;sek-1* animals in liquid pathogen-free media after feeding for 2 h on C. albicans mutants with disruptions in *kem1* (strain MLR74) or *suv3* (strain GKO443). *p* = 0.0008 and *p* < 0.0001, respectively, for the mutants compared to the parental strain C. albicans DAY185 (∼65 nematodes/group).

To evaluate the hypothesis that biofilm formation and filamentation are involved in C. elegans killing, we infected C. elegans with *C. albicans kem1* and *suv3* mutants that are defective in hyphal production [24]. As shown in Figure 3, C. elegans killing by *kem1* (strain MLR74) and *suv3* (strain GKO443) mutants was significantly attenuated compared to the isogenic parental strain DAY185 (*p* = 0.0008 and *p* < 0.0001, respectively for the two mutants, compared to the wild-type strain DAY185). There was no significant difference in killing between the wild-type and strains in which the *kem1* and *suv3* mutations had been reconstituted with wild-type alleles by homologous recombination (unpublished data). Notably, it appears that the interface between solid and liquid is important for filamentation, as it is not observed during *Candida*–C. elegans interaction on solid media (unpublished data and [25]). Because the C. albicans biofilm defective mutants that were used in our studies were also defective in filamentation, the study of the role of *Candida* biofilm in C. elegans pathogenesis requires further experimentation.

**Figure 4:**
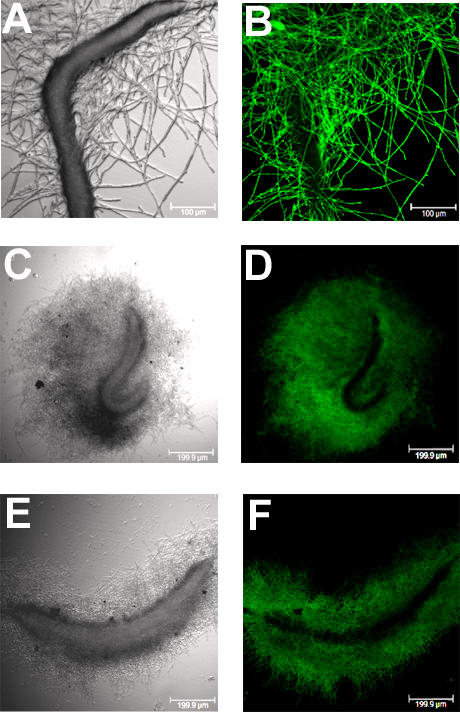
Progression of Filamentation The images show *C. elegans glp-4;sek-1* nematodes infected by C. albicans strain DAY185 for 2 h and then moved into pathogen-free liquid media. On day 1, dead worms were moved to fresh wells containing liquid media and allowed to incubate until day 6 (A and B), or day 8 (C–F). (A, C, and E) are the light-field images and (B, D, and F) are fluorescent images that show the filaments stained with Concanavalin A-Alexafluor, which binds to polysaccharides. Scale bars are 100 μm (A and B) and 199.9 μm (C–F).

Interestingly, in the absence of *C. elegans,* none of the *Candida* species tested in the liquid assay formed filaments. Nevertheless, after nematodes died, extensive filamentation was observed in the colonized worms with numerous filaments exiting through the nematode cuticle (Figure 4A and 4B). Moreover, after the integrity of the cuticle was breached, the yeast cells did not dissipate within the liquid medium, but rather remained connected, resulting in C. elegans “ghosts” in which the transition from mostly yeast-like *Candida* cells to the filaments outlined where the cuticles used to be (Figure 4C–4F).

**Figure 5:**
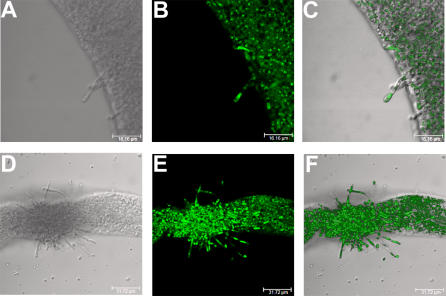
Filamentation Involves the Formation of True Hyphae Fluorescent microscopy of *C. elegans glp-4;sek-1* nematodes after feeding for 2 h on C. albicans strain HGFP3 transformed with pHWP1GFP3 that expresses green fluorescence in a hyphae-specific manner. Worms were transferred to *Candida*-free liquid media for 2 d (A–C) or 6 d (D–F). Green fluorescence is seen in hyphal cells coming out of the nematode as they penetrate the nematode cuticle. Fluorescence is shown in (B and E). (C and F) show overlap images. Scale bars are 16.16 μm (A–C) and 31.72 μm (D–F).

We used a C. albicans reporter strain that expresses yeast-enhanced GFP (yEGFP3) from the promoter for the gene Hyphal Wall Protein 1 *(HWP1)* to evaluate whether true hyphae are formed during the infectious process in *C. elegans.* This strain confers developmental expression of yEGFP3 in germ tubes and hyphae, but not in yeasts or pseudohyphae [18,26,27]. As shown in Figure 5, yEGFP3 expression was observed in filaments as they penetrated the nematode cuticle, confirming that C. albicans forms hyphae in the course of C. elegans infection. No yEGFP3 expression was detected prior to filamentation.

### Antifungals Prolong Nematode Survival

**Figure 6:**
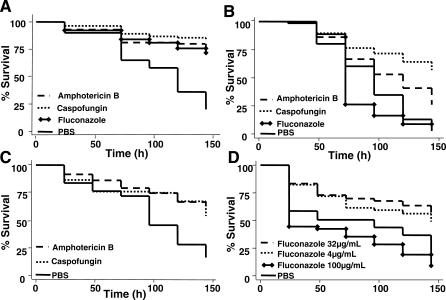
Antifungals Prolong the Survival of *C. elegans glp-4;sek-1* Nematodes Infected by Candida spp. and Evaluation of Antifungals in the C. elegans Assay Allows the Concomitant Evaluation of Toxicity Nematodes were infected with *Candida* for 2 h and then moved to pathogen-free liquid media in the presence of antifungals or PBS. Dead worms were counted and removed daily. (A) C. albicans strain MLR6. *p* < 0.0001 for caspofungin (8 μg/ml), amphotericin B (16 μg/ml), and fluconazole (32 μg/ml). (B) C. krusei strain ATCC#6258. *p* < 0.0001 for caspofungin (8 μg/ml) and amphotericin B (16 μg/ml). (C) C. parapsilosis ATCC#20019. *p* < 0.0001 for caspofungin (8 μg/ml), amphotericin B (16 μg/ml), and fluconazole (32 μg/ml). (D) C. parapsilosis ATCC#20019. The *p-*values are 0.0001 for 4 μg/mL, <0.0001 for 32 μg/mL, 0.01 for 100 μg/mL (∼65 nematodes/group).

To develop a system that allows high-throughput screening for antifungal compounds, we first evaluated whether established antifungals prolong the survival of nematodes infected with Candida spp. For this, we used different C. albicans isolates as well as non-*albicans* strains, including those used as reference strains by clinical microbiology laboratories [28]. Notably, all strains tested were susceptible to amphotericin B and caspofungin, but had different susceptibilities to fluconazole [24,26,29,30] (Table 1). We found that antifungals that are active against a particular *Candida* strain resulted in prolongation of C. elegans survival when the nematodes were infected with the relevant *Candida* strain (Figure 6A–6D). Caspofungin, a fungicidal agent that is active against *Candida* biofilm [31,32], and also the fungicidal agent amphotericin B, were the most active agents tested in prolonging nematode survival to C. albicans strain MLR62 (Figure 6A), C. krusei American Type Culture Collection (ATCC) #6258 (Figure 6B), C. parapsilosis ATCC#20019 (Figure 6C and 6D), and C. albicans ATCC#90028 (unpublished data). In the case of C. krusei ATCC#6258, the most resistant to fluconazole among the strains we tested, caspofungin and amphotericin B were more effective than fluconazole (in both cases *p* < 0.0001; Figure 6B). Antifungal agents were also effective at lower concentrations; for example, caspofungin was effective at concentrations as low as 1 μg/ml in the case of C. albicans strain MLR62 (*p* < 0.0001). Since filament formation was only seen after the *Candida* cells overwhelmed and killed the nematode, there were significantly fewer nematodes with filaments in the wells that contained antifungal compounds (unpublished data).

**Table 1:**
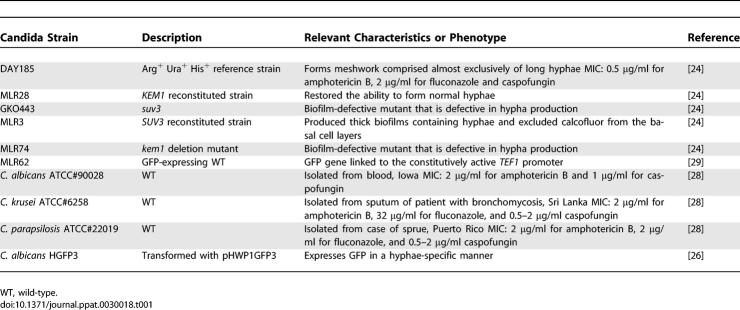
C. albicans Strains Used in This Study

Interestingly, at high concentrations, the beneficial effect of fluconazole was lost and the nematodes died faster than untreated infected worms. Fluconazole up to 32 μg/ml was effective in prolonging survival of nematodes exposed to the fluconazole-susceptible strain *C. parapsilosis* ATCC#20019, but at higher concentrations (100 μg/ml) nematode survival was diminished, even compared to the nematodes in the untreated control group (*p* = 0.01; Figure 6D). Probably this toxicity is present at even lower concentrations, but the beneficial effect from the antifungal activity outweighs the toxic effect. For example, when nematodes were exposed to the fluconazole-resistant strain C. krusei ATCC#6258, fluconazole at 32 μg/ml not only had no effect on nematode survival, but killing in the fluconazole group was significantly faster than untreated worms (*p* = 0.02; Figure 6B). Taken together, these observations suggest that the C. elegans–*Candida* model can be used for the concurrent screen for antifungal activity and host toxicity.

We also evaluated the effect of antifungal agents in reducing fungal burden in the nematode intestine. For example, in a representative experiment, after 48 h, untreated C. elegans infected with C. albicans MLR62 had an average of 170.8 ± 63.5 colony-forming units (cfu) per worm, whereas caspofungin-treated animals (at 8 μg/ml) had almost cleared the infection with an average of 0.3 ± 0.2 cfu per worm (*p* < 0.001). Fluconazole and amphotericin B also significantly decreased the concentration of *Candida* cfu in the nematodes, and microscopic examination of the worms confirmed that treatment with antifungals was effective in clearing the worm intestine of visible *Candida* cells (unpublished data).

### Antifungal Compounds Identified Using the C. elegans–*Candida*-Killing Assay

**Figure 7:**
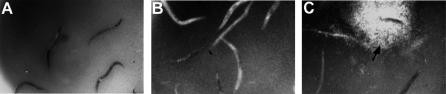
Phenotypic Assay Detects Compounds with Antifungal Activity After exposure to strain C. albicans MLR62, *C. elegans glp-4;sek-1* nematodes were pipetted into 96-well plates that contained compounds from chemical libraries. Nematodes exposed to compounds that had antifungal efficacy (in this case, CAPE) had no fluorescence within the intestine (A), whereas nematodes exposed to compounds without antifungal efficacy did not demonstrate any movement (B) and developed filaments outside the nematodes (C). Roughly 25 worms were used per well.

To facilitate adaptation of the C. elegans–*Candida-*killing assay to screening chemical libraries for antifungal compounds, we used C. albicans strain MLR62 that expresses GFP (linked to the constitutively active *TEF1* promoter) and that exhibits similar killing kinetics to the parent strain DAY185 in the C. elegans assay. Using this strain, nematodes exposed to compounds that have significant antifungal efficacy exhibited sinusoidal movement and no green fluorescence in the intestine at the endpoint of the assay (Figure 7A), whereas nematodes exposed to compounds without antifungal efficacy did not move, were rod-shaped, exhibited high levels of intestinal fluorescence (Figure 7B), and developed filaments (Figure 7C).

As an initial test of the C. elegans–*Candida* compound screening assay, we utilized libraries of compounds made available through the Institute of Chemistry and Cell Biology (ICCB) at Harvard Medical School that include known compounds that affect diverse cellular pathways as well as the United States Food and Drug Administration approved drugs that are known to be safe and bioactive in humans (http://iccb.med.harvard.edu). We screened a total of 1,266 compounds and identified 15 (∼1.2%) that prolonged nematode survival and completely or almost completely inhibited filamentation (Table S1). An additional 57 (∼4.5%) compounds prolonged nematode survival but had no or minimal effect on filamentation (Table S1). To the best of our knowledge, the C. elegans screen identified all of the compounds among the 1,266 tested with established, clinically documented antifungal activity against *Candida* (Table S1)*.* Among the known antifungals, ketoconazole and butoconazole were among the most effective compounds identified (Table S1).

**Figure 8:**
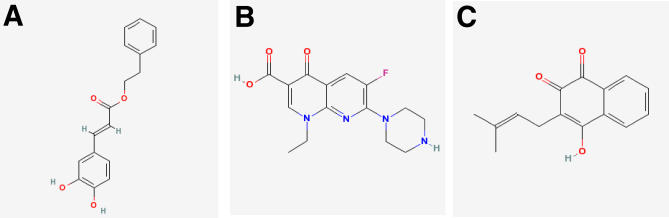
Chemical Compounds Identified Using the *C. elegans–Candida* Screen Tested in a Murine Model of Hematogenous Candidiasis (A) CAPE, (B) enoxacin, and (C) lapachol (from http://pubchem.ncbi.nlm.nih.gov).

We selected three compounds identified in the screen, which are not established antifungals, for further analysis. By design, we selected compounds that have a positive safety profile in mammals and exhibit variable efficacy in the C. elegans assay, including compounds that both affected or did not affect filamentation. The three compounds selected for further evaluation were caffeic acid phenethyl ester (CAPE), enoxacin, and lapachol (Figure 8 and Table S1).

CAPE is a major active component of propolis, a natural antimicrobial resinous product derived from plants, which is collected and used by honeybees to seal cracks or large potentially infectious objects (such as carcasses) that honeybees cannot remove from their hives ([33,34] and Table S1). Enoxacin, a fluoroquinolone antibiotic, was shown previously to marginally enhance the in vitro antifungal activity of amphotericin B and mepartricin [35]. Lapachol is a naphthoquinone found in the seeds and heartwood of tropical plants that exhibits anti-leishmanicidal activity [36–38]. Enoxacin did not inhibit C. albicans filament formation in the C. elegans-killing assay.

**Figure 9:**
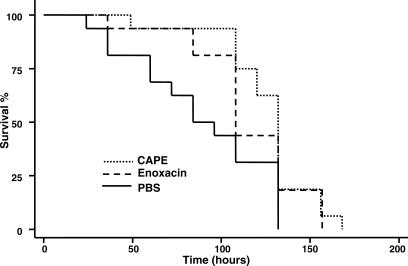
Compounds Identified in the C. elegans Screen Have Activity in a Murine Model of Hematogenous Candidiasis Survival of mice after tail vein injection of 1 × 10^6^ blastospores of C. albicans DAY185. Mice that received intravenous injection of CAPE (400 mg/kg once as a loading dose, followed by 200 mg/kg maintenance twice daily) or enoxacin (80 mg/kg loading dose, followed by 40 mg/kg maintenance twice daily) survived significantly longer compared to control mice that received placebo (*p* = 0.008 and 0.04, respectively). There were 16 mice in each group.

To determine whether CAPE, enoxacin, and lapachol play a role in mammalian virulence, we evaluated their efficacy in an established murine model of systemic *Candida* infection. After tail vein injection of 1 × 10^6^ blastospores, both CAPE and enoxacin prolonged the survival of mice inoculated with C. albicans strain DAY185 (Figure 9). Among these three compounds, CAPE, which was the most effective agent in the C. elegans screen, was also the most effective agent in the murine assays (*p* = 0.008, compared to the control of mice that received placebo). Notably, intravenous enoxacin was effective in once-a-day dosing of 25 mg/kg (*p* = 0.03; 12 mice per group), whereas CAPE was not statistically effective in once-a-day dosing (unpublished data). Lapachol (150 mg/kg) was not active in the mouse model (unpublished data), consistent with previous work that suggested that lapachol may be transformed into inactive metabolite(s) or neutralized in mammals [36–38].

**Figure 10:**
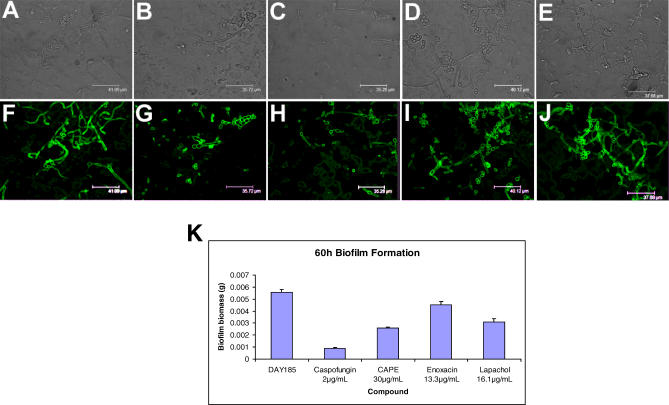
In Vitro Efficacy of CAPE, Enoxacin, and Lapachol in Biofilm Formation DAY185 formed biofilms on silicone squares in the presence of DMSO in PBS (A and F), caspofungin (B and G), CAPE (C and H), enoxacin (D and I), or lapachol (E and J). Silicone squares were observed for filament formation with a 40× oil emersion objective (A–E) and stained with concanavalin A-Alexa 488 to identify the cell membranes of DAY185 with confocal microscopy (F–J). All three compounds significantly decreased the dry mass of the silicone pads (*p* = 0.020 for caspofungin, CAPE, and lapachol; and *p* = 0.029 for enoxacin compared to DMSO in PBS control) (K).

All three compounds were also evaluated in vitro for their activity in *Candida* biofilm formation. CAPE strongly inhibited both filamentation and biofilm formation (Figures 10 and S2 and Text S1). Interestingly, lapachol also exhibited a modest effect on biofilm formation, suggesting that metabolites of lapachol that are not transformed into inactive metabolite(s) or neutralized in mammals [36–38] may be useful antifungal agents. Of note is that the antifungal activity of the three compounds tested is most likely not due to their ability to inhibit the growth of yeast. Among the three compounds, only CAPE inhibited growth of C. albicans in vitro, and that was at a minimum inhibitory concentration (MIC) of 64 μg/ml, which was higher than the concentration (33 μg/ml) used in the C. elegans screen. Moreover, CAPE was effective in the C. elegans–*Candida* assay to a concentration as low as 4 μg/ml (unpublished data). Enoxacin and lapachol did not inhibit the growth of C. albicans strain DAY185 at the highest concentration tested (256 μg/ml).

To evaluate for toxicity, CAPE, enoxacin, and lapachol were evaluated in vitro using sheep erythrocytes [7] and in vivo using *glp-4;sek-1* worms in liquid media with E. coli OP50. Neither CAPE nor lapachol hemolyzed the red blood cells, as compared to DMSO and Triton X-100 controls, though enoxacin exhibited a marginal amount of hemolysis; however, none of the three compounds had any acute toxicity to uninfected *C. elegans glp-4;sek-1* worms (unpublished data).

## Discussion

The objective of the studies detailed in this paper was to develop a semi-automated, high-throughput, whole-animal C. elegans assay that can be used to identify chemical compounds with antifungal activity. Key features of the C. elegans assay are that it allows concurrent evaluation of toxicity and antifungal activity and that it identifies compounds that target important pathways associated with human *Candida* infection, including filament formation. Moreover, the assay allows the study of *Candida* cells that are in non-planktonic form and identification of antifungal compounds in a system where both the pathogen and the host can be genetically manipulated.

The C. elegans whole-animal screen has several advantages compared to in vitro screens that use planktonic cells. Some virulence traits are induced only in the host, and therefore the identification of compounds that are effective against these virulence traits may require detection in vivo. Also, the whole-animal approach provides two relatively unambiguous assay endpoints in the survival/death of the worms and filamentation, and allows the use of liquid-handling robots for filling assay plates and for pin transfer of compounds from library stock plates to assay plates. The current rate-limiting step in the assay is the manual scoring of worm viability and filament formation. Thus far, scoring of live/dead and filamented/nonfilamented worms has been done by direct human observation of movement and morphology of the nematodes on a microscope or by reviewing microscopic images. A quantifiable automated scoring assay that is compatible with the use of high-throughput automated screening microscopes is currently under development and would greatly increase the throughput of the assay. Also, the delivery of a standard number of worms in each well would further enhance the uniformity of the assay. This is possible using a commercially available worm-dispensing robot. Moreover, in the future, we believe that it will be possible to robotically dispense both *Candida* and C. elegans to the wells, thereby eliminating the labor-intensive infection and wash steps.

The C. elegans assay has many advantages compared to screens using mammalian models. The study of pathogenesis in mammals is complicated by difficulty of handling, long reproductive cycles, small brood sizes, complexity of mammalian hosts, high cost, and ethical considerations. In vivo models for *Candida* biofilm are particularly cumbersome and expensive, as they usually entail the placement of vascular catheters in rabbits [39] or rats [40,41]. Overall, the whole-animal C. elegans screen can help identify compounds that most likely would not be identified by in vitro screens or would be too costly and inefficient using an in vivo mammal approach. In addition to the *Candida* assay, members of our group have screened libraries of chemical compounds to identify those that have antimicrobial activity against C. elegans that are persistently infected with the human opportunistic pathogen Enterococcus faecalis [7].

However, it should be noted that the use of C. elegans for the identification of antimicrobial agents also has obvious limitations. First, it is unlikely that the use of C. elegans (or other invertebrate model hosts) will completely eliminate toxic compounds, even though there is a reasonable correlation between the toxicities of a variety of compounds in mammals and C. elegans [7,42–44]. In addition, the small size that makes C. elegans less expensive and amenable to high-throughput screens makes it very difficult to use this model to study pharmacokinetic variables such as impact of protein binding, absorption, and distribution that are automatically assayed in other, larger animal models.

In addition to providing a facile system for screening chemical libraries for antifungal compounds, the experiments detailed above support the hypothesis that natural selection in nonmammalian hosts may have aided the evolution and maintenance of virulence-related genes in pathogenic human fungi [8,45]. In previous work from our laboratories, we showed that *Cryptococcus neoformans,* another important human opportunistic fungal pathogen, is also a potent C. elegans pathogen, supporting previous conclusions that the pathogenic potential of C. neoformans may have evolved as a by-product of avoiding predation by soil organisms, including nematodes and amoebae that feed on yeasts [46]. Thus, even though C. albicans is not necessarily found in the same environment as *C. elegans,* other fungal pathogens are found in the soil, and the interactions between C. elegans and fungi in general can be seen as a representation of the evolutionary arms race between fungi and soil-borne predators such as C. elegans [47]. Interestingly, however, C. neoformans and *Candida* use different approaches to kill *C. elegans.* Cryptococcal cells, which are larger than *Candida* cells, do not persist in the nematode intestine, and nematodes exposed to cryptococcal lawns are able to defecate the cryptococcal cells upon transfer to liquid media and thus can clear the cryptococcal infection ([8] and unpublished data). In contrast, *Candida,* a common colonizer of the intestinal track of humans, establishes a persistent infection in the C. elegans intestine, forms biofilm, dissolves nematode tissues, and breaks through the nematode cuticle by forming an impressive network of filaments (Figures 2, 4, and 5).

It is expected that the C. elegans–*Candida*-killing assay will not only identify compounds that affect the growth of the pathogen in vitro, but also compounds that affect the virulence of the pathogen in vivo, or, even immuno-modulate the host. For example, CAPE is known to inhibit NfkappaB [48] in mammals, and murine mouse studies suggest that it has immunomodulatory effects in vivo [49,50]. Since C. elegans appears not to have an NF-kB-like protein, if CAPE also affects C. elegans immunity, the mechanism presumably does not involve NF-kB inhibition. Since the compounds identified through this screen have no obvious similarities with current classes of antifungals and thus may have a different mechanism of action, combination of these compounds with established antifungal agents could have additive or synergistic action that should be studied further.

## Materials and Methods

### Strains and media.

The *Candida* strains used in these experiments are summarized in Table 1 or described in the text. Yeast cultures were maintained on yeast peptone dextrose (YPD) (Difco, http://www.vgdusa.com/DIFCO.htm), agar, or as frozen stocks. The C. elegans strains were propagated on *E*. *coli* strain OP50 or E. coli strain HB101 using established procedures (detailed below and in [7,51]).

### C. elegans liquid medium killing assays.

*Candida* strains were inoculated in 2 ml of YPD and grown at 30 °C for 24 h. Lawns were prepared by spreading 10 μl of each culture on 35-mm tissue culture plates (BDFalcon, http://www.bdbiosciences.com) containing solid BHI media (Difco) with kanamycin (45 μg/ml), ampicillin (100 μg/ml), and streptomycin (100 μg/ml). The plates were incubated at 30 °C for 24 h followed by 25 °C for 24 h. *glp-4;sek-1* and *glp-4* worms were grown at 15 °C until they reached the L2 stage, and then they were transferred to 25 °C for 24 h. N2 wild-type worms were grown at 15 °C for 72 h, until they reached the L4 stage. Approximately 100 worms were picked onto each lawn and allowed to feed for 30 min to 2 h. The worms were washed off the plates with M9 buffer and allowed to crawl on unseeded BHI plates to remove yeast cells from their cuticles. Roughly 70–80 worms were then picked to wells in a six-well microtiter dish that contained 1.5 ml liquid medium of 79% M9 buffer, 20% BHI, 10 μg/ml cholesterol in ethanol, and 90 μg/ml kanamycin. The plates were incubated at 25 °C overnight and then examined at 24-h intervals for survival. Worms were considered dead and removed when they did not respond to being touched by a platinum wire pick.

### C. elegans filamentation staining.

C. albicans strains DAY185 and HGFP3 lawns were prepared, and *C. elegans glp-4;sek-1* worms exposed to C. albicans as described above for the killing assays. The worms were then transferred to the liquid media described above in both the presence and absence of 32 μg/ml fluconazole. At 24, 48, 144, and 192 h, 20–40 worms exposed to DAY185 were stained in 200 μl of 10 μM FUN-1 (Invitrogen, http://www.invitrogen.com) and 25 μg/ml Concavalin A-Alexafluor 488 (Invitrogen) for 45 min [52]. Pictures were taken with a confocal laser microscope (TCS NT, Leica Microsystems, http://www.leica-microsystems.com). FUN-1 is a fluorescent yellow dye that is absorbed by metabolically active fungal cells and fluoresces red when illuminated with 480 nm (fluorescence emission). Concanavalin A-Alexafluor (fluorescence emission at 519 nm) is a fluorescent green dye that binds to polysaccharides and stains filaments.

### Evaluation of *Candida* cells within the nematode intestine.

MLR62 and DAY185 lawns were prepared as above. An OP50-GFP strain was grown in LB media in the presence of ampicillin (100 μg/ml), instead of streptomycin and spotted on nematode growth media (NGM) agar plates. For the first experiment, *sek-1;glp-4* worms were exposed to MLR62 for 1 h, washed thoroughly, and then pipetted onto DAY185 for 2 h. The worms were then moved to pathogen-free liquid media and photographed on days 0 and 1 with the confocal laser microscope mentioned above. For the second experiment, *sek-1;glp-4* worms were placed on DAY185 for 2 h, thoroughly washed, and then pipetted onto OP50-GFP NGM plates. Worms were thoroughly washed and then photographed with a confocal laser microscope at 2, 24, and 48 h. Additionally, living and dead worms on day 1 were moved to pathogen-free liquid media in the presence of kanamycin (90 μg/ml) and monitored for hyphae formation.

### Evaluation of fugal burden within *C. elegans.*

The number of C. albicans cfu in C. elegans was quantified based on the protocol detailed in [45,53] with a few modifications. C. albicans strain MLR62 lawns were prepared on BHI as described above. *C. elegans glp-4;sek-1* worms were exposed to the lawns for 30 min and then moved to liquid media and incubated at 25 °C in the presence or absence of antifungal agents. At appropriate time points, three groups of ten worms each were washed twice in 8-μl drops of M9 medium on BHI agar plates, in order to remove surface *Candida.* Each group of ten worms was then disrupted using a Pellet Pestle Motor (Kontes, http://www.kimble-kontes.com) and plated on YPD agar containing kanamycin (45 μg/ml), ampicillin (100 μg/ml), and streptomycin (100 μg/ml). The plates were incubated for 48 h at 30 °C and colonies were counted. Statistical significance was determined by the Mann-Whitney test using STATA6 statistical software (Stata, http://www.stata.com). A *p-*value of less than 0.05 was considered to be significant.

### Screen of compound library in C. elegans.

For testing the efficacy of chemical compounds, C. albicans strain MLR62 was inoculated into 2 ml of YPD and grown at 30 °C for 24 h; 10 μl of the culture was spread on 35-mm tissue culture plates (BDFalcon) containing BHI agar (Difco) with kanamycin (45 μg/ml), ampicillin (100 μg/ml), and streptomycin (100 μg/ml). The plates were incubated at 30 °C for 24 h. C. elegans animals at the L4 developmental stage (grown as detailed above under C. elegans liquid medium killing assays) were transferred from a lawn of E. coli HB101 onto C. albicans lawns on BHI medium, incubated at 25 °C for 30 min, and then pipetted in 50 μl into 96-well plate wells that contained 100 μl liquid media (79% M9 buffer, 20% BHI media, 10 μg/ml cholesterol in ethanol, and 90 μg/ml kanamycin). *C. elegans glp-4;sek-1* nematodes were pipetted into 96-well plates that contained compounds from chemical libraries. Library 1361 was assembled by Biomol. It contains well-characterized compounds that affect many different aspects of cellular pathways. The NINDS custom collection (plates 501–503) was put together by MicroSource Discovery Systems for the National Institute of Neurological Disorders and Stroke (NINDS), the Huntington Disease Society of America (HDSA), the Amyotrophic Lateral Sclerosis (ALS) Association, and the Hereditary Disease Foundation (HDF). It mostly contains FDA-approved drugs. The Prestwick library (plate 1569) contains compounds that are known to be safe and bioactive in humans. The majority of the compounds are marketed drugs (details available at http://iccb.med.harvard.edu).

On day 6, nematodes were evaluated for survival, filament formation, and optical density. The plates were incubated at 25 °C and examined on day 6 for survival with a Nikon SMZ645 dissecting microscope, and images were obtained using a CellWorx High Content Cell Analysis System (Applied Precision, http://www.appliedprecision.com) at 4× magnification. Dead worms were counted but not removed. Worms were considered dead if they had developed filamentation, were rod-shaped, or did not respond to the well being tapped. Each compound was also scored by the number of dead worms in the well that developed hyphea (Table S1). Compounds were considered to have completely or almost completely inhibited filament formation if fewer than 25% of the dead worms in the well developed hyphea. Compounds were categorized as having a minimal effect on filament formation if 25%–75% of the dead worms developed hyphea. The compounds that had no effect on filament formation allowed over 75% of the dead worms to form hyphea.

Approximately 25 nematodes were used to analyze each chemical compound tested. Two controls were used in all plates. The positive control was the antifungal caspofungin (which allows >75% survival at day 6). Phosphate-buffered saline (PBS) was the negative control (<25% of nematodes in PBS are alive at day 6). In this pilot screen, we considered a compound as a “hit” when survival in the compound well was 50% and above of the median survival of the control wells.

### Evaluation of MIC.

Cells were maintained in YPD (1% yeast extract, 2% peptone, and 2% dextrose) at −80 °C until used. The MIC was evaluated for each of the above compounds according to the Clinical and Laboratory Standards Institute (formerly the National Committee for Clinical Laboratory Standards, NCCLS) microdilution protocol (M27-A) [28].

### Hemolysis assay.

CAPE, enoxacin, and lapachol were evaluated for hemolysis of sheep erythrocytes as described by Moy et al. [7]. DMSO and Triton X-100 were used as controls.

### Compound toxicity in uninfected *glp-4;sek-1* worms.

To test whether CAPE, enoxacin, and lapachol were toxic to the worms at the concentration used in the hemolysis assay, *glp-4l;sek-1* L4s were prepared as described above. The nematodes were moved from E. coli OP50 to pathogen-free liquid media containing 2% DMSO or 2% Triton X-100 or 100 μg/ml CAPE, enoxacin, or lapachol. The worms were incubated for 4 h at 25 °C; at hourly intervals dead worms were counted and removed.

### Murine models of virulence.

BALB/c mice (22–24 g, female, Charles River Laboratories) were used in all studies. In brief, C. albicans strain DAY185 was grown at 30 °C with shaking overnight in YPD to late-log phase. The yeast cells were centrifuged, washed in PBS, and re-suspended to a concentration of 5 × 10^6^ blastospores/ml (measured with a hemocytometer). 200 μl of this suspension were inoculated into the lateral tail vein (1 × 10^6^ blastospores). In all mammalian experiments, the concentration of yeast cells in the inoculum was confirmed by plating serial dilutions and enumerating cfus 24–48 h later. The murine protocols were approved by the Massachusetts General Hospital Committee on Research, Subcommittee on Research Animal Care and special attention was given to minimize the suffering of the mice.

### In vitro biofilm growth on silicone pads.

The effect of CAPE, lapachol, and enoxacin on biofilm growth on silicone pads was evaluated as described by Richard et al. [24] and Nobile et al. [41]. The C. albicans strain DAY185 was inoculated in 2 mL of YPD and grown at 30 °C for 24 h. The DAY185 culture was diluted into SD media plus 50 mM glucose to an OD_600_ of 1.0. After the 90-min incubation and PBS wash, caspofungin (positive control), CAPE, enoxacin, and lapachol were added to fresh media at 2 μg/mL, 30 μg/mL, 13.3 μg/mL, and 16.1 μg/mL, respectively.

### Microscopic visualization of silicone pads.

The silicone pads were visualized as reported in [24,29]. DAY185 cells were incubated at 37 °C for 60 h on silicone squares in SD media with 50 mM glucose. Biofilms were stained with 25 μg/ml Concavalin A-Alexafluor 488 (Invitrogen) for 1 h in the dark. Pictures were taken with a confocal laser microscope (TCS NT, Leica Microsystems).

### Biofilm dry mass measurements.

The biofilm dry mass was evaluated as described by Nobile et al. [29]. Statistical significance was determined by the Mann-Whitney test using STATA 6. A *p-*value of less than 0.05 was considered to be significant.

### Statistical analysis.

Murine and C. elegans-killing curves were plotted, and estimation of differences (log-rank and Wilcoxon tests) in survival analyzed with the Kaplan-Meier method performed using STATA 6. A *p-*value of less than 0.05 was considered to be significant.

## Supporting Information

Figure S1 *Candida* Species Accumulate in the C. elegans Intestine (A) C. elegans nematode strain *glp-4;sek-1* exposed to C. parapsilosis strain ATCC# 22019 for 2 h and then moved to microbe-free liquid media. 1 d later, there were a significant number of yeast cells within the nematode resulting in distention of the nematode intestine. The round structure (white arrows) is the pharyngeal grinder organ, which functions to disrupt ingested organisms. Black arrows with borders point to the intestinal lumen.
(B) As the infection within the nematode progresses, the nematode cells are essentially replaced by yeast cells, and staining of nematodes with FUN-1 shows yeast cells to be metabolically active (*C. elegans glp-4;sek-1* nematode 4 d after infection with C. parapsilosis strain ATCC#22019).
(1.6 MB TIF)

Figure S2 In Vitro Efficacy of CAPE, Enoxacin, and Lapachol Biofilm on the Surfaces of Wells of Microtiter Plates When evaluated using the XTT reduction assay, CAPE and lapachol inhibited the formation of biofilm (A), while all three compounds had an affect in pre-formed biofilm (B).
(58 KB TIF)

Table S1 Compounds Identified through a Pilot Screen in C. elegans
(349 KB DOC)

Text S1 Supplemental Methods and Reference (22 KB DOC)

### Accession Numbers

The protein sequences from the TrEMBL database (http://www.ebi.ac.uk/trembl) discussed in this paper are HWP1 (Q59TL7) and SEK-1 (Q95Y19).

## Abbreviations

ATCC: American Type Culture Collection
BHI: brain–heart infusion
CAPE: caffeic acid phenethyl ester
cfu: colony-forming unit
GFP: green fluorescent protein
MIC: minimum inhibitory concentration
PBS: phosphate-buffered saline, YPD, yeast-peptone dextrose
