# Supplementary material for: Antifungal Chemical Compounds Identified Using a C. elegans Pathogenicity Assay
Source: PLoS Pathog. 2007 Feb 2;3(2):e18. doi: 10.1371/journal.ppat.0030018 (PMC1790726; doi:10.1371/journal.ppat.0030018)
Supplement: Supplementary file 1 [file ppat.0030018.sd001.doc]

**Text S1.**

**Figure S1.** (**a**) *C. elegans* nematode strain *glp-4;sek-1* exposed to *C. parapsilosis* strain ATCC# 22019 for 2 hours and then moved to microbe-free liquid media. A day later, there was a significant number of yeast cells within the nematode resulting in distention of the nematode intestine. The round structure (white arrows) is the pharyngeal grinder organ, which functions to disrupt ingested organisms. Black arrows with borders point to the intestinal lumen. (**b**) As the infection within the nematode progresses the nematode cells are essentially replaced by yeast cells and staining of nematodes with FUN-1 shows yeast cells to be metabolically active (*C. elegans glp-4;sek-1* nematode four days after infection with *C. parapsilosis* strain ATCC#22019).

**Figure S2. *In vitro* efficacy of CAPE, enoxacin and lapachol biofilm on the surfaces of wells of microtiter plates.** When evaluated using the XTT reduction assay CAPE and lapachol inhibited the formation of biofilm **(a)**, while all three compounds had an affect in pre-formed biofilm (**b**).

Supplemental Methods

**Effect of compounds on adherent cells and subsequent biofilm formation on the surfaces of wells of microtiter plates.** The effect of the compounds on adherent cells and subsequent biofilm formation was evaluated using the XTT reduction assay described by in [106]. The *C. albicans* strains DAY185 was inoculated in 2mL of RPMI 1640 and grown at 30ºC for 24h. Working concentrations of each compound were prepared in RPMI 1640. DMSO and dH_2_O were diluted with RPMI 1640 and used as controls.

**Effect of compounds on preformed biofilms on the surfaces of wells of microtiter plates.** The effect of compounds on preformed biofilms were evaluated as described in [106]. *C. albicans* biofilms were formed for 24h at 37°C on the surfaces of microtiter plates by using the protocol described above. After the biofilms were formed, the medium was aspirated, and non-adherent cells were removed by washing the biofilms three times in sterile PBS. Compounds were added into selected wells of the microtiter plates and plates were then incubated for 24 h at 37°C. The effect of each compound on preformed biofilms was then estimated by using the XTT reduction assay (above).

**Supplemental References**

106. Ramage G, Saville SP, Wickes BL, Lopez-Ribot JL (2002) Inhibition of *Candida albicans* biofilm formation by farnesol, a quorum-sensing molecule. Appl Environ Microbiol 68: 5459-5463.
