# Supplementary material for: Antifungal Chemical Compounds Identified Using a C. elegans Pathogenicity Assay
Source: PLoS Pathog. 2007 Feb 2;3(2):e18. doi: 10.1371/journal.ppat.0030018 (PMC1790726; doi:10.1371/journal.ppat.0030018)
Supplement: Supplementary file 2 [file ppat.0030018.st001.doc]

**Table S1. Compounds that completely or almost completely inhibited filament formation.**

| Compo  und | Action/Use | Killing* | **Reference(s)** |
| --- | --- | --- | --- |
| Caffeic acid phenethyl ester | NFkappaB inhibitor which plays important role in transcription factors pertinent to interleukin 1 action | 0 | [1] |
| Ketoconazole | Antifungal | 0.056, 0.167 | [2] |
| AG-370 | Belongs to tyrphostin family of tyrosine kinase inhibitors. Inhibits mitogenesis in human fibroblast mediated by platelet derived growth factor | 0.111 | [3] |
| Butoconazole nitrate | Antifungal | 0.128 | [4] |
| Methscopolamine bromide | Anticholinergic; blocks M1 muscarinic receptors who are present in *Saccharomyces cerevisiae* | 0.167 | [5-7] |
| Lapachol | Antibacterial/Antiparasitic | 0.182 | [8,9] |
| Diethylstilbestrol | Synthetic estrogen; estrogens stimulate vagina colonization with *Candida albicans* | 0.235 | [10,11] |
| Probucol | Lipid-lowering agent/Antioxidant | 0.278 | [12] |
| Sulfabenzamide | Antibiotic | 0.286 | [13] |
| Prochlorperazine edisylate | Antiemetic/Antipsychotic; antibacterial activity *in vitro* | 0.300 | [14-16] |
| Budesonide | Glucocorticoid | 0.385 | [17] |
| Cephaloridine | Antibiotic | 0.385 | [18] |
| Dyclonine hydrochloride | Local anesthetic; inhibits ergosterol synthesis | 0.444 | [19,20] |
| Flutrimazole | Antifungal | 0.444 | [21] |
| Hydroxytacrine maleate | Metabolite of tacrine; tacrine is an acetylcholinesterase inhibitor that is used against Alzheimer’s disease; reduces apoptosis in neuronal cells | 0.467 | [22] |

Compounds that had minimal effect on filament formation.

| 5 (S) HPETE | Ca^2+^ channel activation resulting in B-lymphocyte activation | 0.261 | [23] |
| --- | --- | --- | --- |
| Ketorolac tromethamine | Anti-inflammatory | 0.267 | [24-26] |
| Nimodipine | Ca^2+^ channel blocker (L-type)/Probable antioxidant action (see methoxyverapamil) | 0.273 | [27,28] |
| Lipoxin A4 | Product of interaction of 15-HPETE with human leukocytes; anti-inflammatory mediator, suppresses inflammation; inhibits NK cell cytotoxicity | 0.273 | [29-31] |
| N-Arachidonoylglycine | Conjugate of lipids and amino acids; suppresses tonic inflammatory pain in bovine and rat brain; produces endocannabinoid-induced inhibition of T-cell proliferation | 0.275 | [32,33] |
| Lidocaine | Na^+^ channel blocker; local anesthetic; antiarrhythmic; has antibacterial activity against *E.coli*, *P.* *aeruginosa*, *S. aureus* *in vitro* and antifungal activity against *C. albicans* | 0.286 | [34,35] |
| L-cis-Diltiazem | Ca^2+^ channel blocker; antioxidant | 0.296 | [36] |
| AG-1478 | Epidermal growth factor inhibitor; antioxidant | 0.3 | [37,38] |
| 7,7-Dimethyleicosadienoic acid | Probable antioxidant activity | 0.304 | [39] |
| MBCQ | Selective inhibitor of cGMP-specific phosphodiesterase | 0.32 | [40] |
| 1-Stearoyl- 2-linoleoyl-glycerol | Probably enhances the effect of TNF alpha | 0.333 | [41] |
| Bafilomycin A1 | Endosomal H(+)-ATPase inhibitor in yeasts; Inhibits TNF-α and expression of metalloproteinases;  reduces killing of *Aspergillus fumigatus* by alveolar macrophages | 0.333 | [42-44] |
| Eicosatrienoic acid (20:3 n-3) | Product of arachidonic acid; induces mitogenesis | 0.333 | [45] |
| Farnesylthioacetic acid | Farnesyl derivative; Ras antagonist | 0.343 | [46] |
| Niflumic acid | NSAID; inhibits growth of *C. albicans* via cytosolic acidification and inhibition of glycolysis | 0.355 | [47] |
| Siguazodan | Phosphodiesterase III inhibitor; inhibits generation of interleukins-4 and -13 | 0.375 | [48,49] |
| Aminobenzamide (3-ABA) [3-aminobenzamide (3-ABA)] | Tx+ poly(ADP-ribose) synthetase inhibitor; inhibits apoptosis in rat intestinal cells; suppresses inflammatory response and brain injury in experimental *E. coli* meningitis | 0.389 | [50]^,^[51] |
| Acetyl (N) s farnesyl l cys | Inhibitor of isoprenylated protein endoprotease resulting in inhibition of function of ras protein | 0.4 | [52,53] |
| Cefmetazole sodium | Chemotactic agent for neutrophils | 0.4 | [54] |
| Glipizide | Sulfonylouria; increases oxidative stress in red cells *in vitro* | 0.4 | [55] |
| Phenylpropanolamine hydrochloride | Catecholamine; inotropic | 0.407 | [56] |
| 15 (S) HPETE | Enhances TNF/cycloheximide induced apoptosis, activates caspases-3 and -9 | 0.412 | [57] |
| Ascorbic acid | Vitamin C; antioxidant; appears to enhance growth of *Cryptococcus neoformans* and *Candida tropicalis in vitro*; enhances neutrophil motility and lymphocyte transformation *in vitro* and *in vivo* after infection with *Candida albicans* | 0.417 | [58-60] |
| FPL-64176 | Induces apoptotic cell death via activation of L-type Ca^2+^ activation | 0.429 | [61] |
| Ikarugamycin | Antiprotozoan/Inhibits degradation of CD4 | 0.429 | [62] |
| SDZ-201106 | Na^+^ channel modulator | 0.432 | [63] |
| Cytochalasin B | Actin polymerization inhibitor | 0.438 | [64] |
| Docosatrienoic acid (22:3 n-3) | Fatty acid; reduces GABA response in rat substantia nigra neurons; is metabolized by fungal lipases to a high-laurate canola oil | 0.439 | [65,66] |
| 3,4-Dichloroisocoumarin | NF-kappaB inhibitor; decreases TNF alpha effect on migration of lymphocytes | 0.444 | [67] |
| ALA-ALA-PHE-CMK | Catalytic residue; probably antioxidant | 0.448 | [68] |
| Eicosapentaenoic acid (20:5 n-3) | Fatty acid; is metabolized by fungal lipases to a high-laurate canola oil | 0.448 | [66] |
| Rolipram | Phosphodiesterase 4A inhibitor; anti-inflammatory; inhibits IL-2R | 0.455 | [69-71] |
| AG-490 | Blocks cell growth and induces apoptosis in human acute lymphocytic leukemia cells; blocks the activity of interleukin-7 in T-lymphocytes | 0.462 | [72-74] |
| Misoprostol, free acid | Prostaglandin; reverses suppression of TGF-β caused by acetyl salicylic acid; activates protein-kinase A resulting in inhibition of HIV-replication into macrophages | 0.464 | [75,76] |
| Betulinic acid | Inhibits secreted aspartic proteinases of *C. albicans* | 0.483 | [77] |
| Leukotriene B4 | Stimulates monocytes, neutrophils and dendritic cell chemotaxis | 0.483 | [78] |
| DL-Dihydrosphingosine | Sphingolipid; Probable antibacterial and antifungal activity via inhibition of cell wall | 0.485 | [79] |
| N-palmitoyl-L-serine phosphoric acid | Induces platelet aggregation | 0.486 | [80] |

Compounds that had no effect on filament formation.

| Butamben | Local anesthetic; contains p-aminobenzoate which decreases the activity of coenzyme Q dependent electron transport | 0.105 | [81,82] |
| --- | --- | --- | --- |
| Pentolinium bitartrate | Inhibitor of catecholamine secretion | 0.227 | [83] |
| Hydrocortisone hemisuccinate | Glucocorticoid | 0.300 | [84] |
| Ambroxol hydrochloride | Na^+^ channel blocker/Pain reliever/Mucolytic agent; promotes microbicidal activity of monocytes; anti-inflammatory activity | 0.303 | [85-87] |
| Clopamide | Thiazide diuretic; enhances oxidative stress | 0.333 | [88,89] |
| Brinzolamide | Antiglaucomatic/Carbonic anhydrase inhibitor; antibacterial | 0.346 | [90] |
| Streptomycin sulfate | Antibiotic | 0.364 | [91] |
| Benfluorex hydrochloride | Insulin sensitizer; activates transcription factors in *Saccharomyces cerevisiae* | 0.375 | [92,93] |
| Enoxacin | Fluoroquinolone; *in vitro* synergy with antifungals | 0.379 | [94] |
| Practolol | Beta-blocker; enhances free radical production by neutrophils | 0.381 | [95] |
| Betamethasone | Glucocorticoid; inhibits glucose-beta-phosphate dehydrogenase, an enzyme present in yeasts | 0.417 | [96] |
| Amoxicillin | Antibacterial; enhances colonization of gastrointestinal tract with *Candida* | 0.429 | [97] |
| Pregnenolone sulfate, sodium salt | Neurosteroid; induces apoptotic cell death on retinal cells via activation of caspase-2 and -3 and cytochrome c release | 0.438 | [98] |
| Clomiphene citrate (Z,E) | Antiestrogen; decreases fatty acid production in *Saccharomyces cerevisiae* | 0.440 | [99] |
| Dobutamine hydrochloride | Inotropic agent; stimulates biofilm formation by *Staphylococcus epidermidis* | 0.455 | [100] |
| Probenecid | Induces apoptosis | 0.457 | [101] |
| Adenosine 5'-monophosphate monohydrate | Induces yeast-mycelium transition in *C. albicans* | 0.471 | [102] |
| Azathymine, 6 | Antimetabolite; a derivative of this compound has antibacterial and antifungal activity | 0.480 | [103,104] |
| Benzbromarone | Increases uric acid excretion from kidneys; Probably inhibits apoptosis of proximal tubular renal cells | 0.483 | [105] |

* The number in the killing column is the number of dead worms divided by the total number of worms in the well

**Supplemental Figures**

**Figure S1.** (**a**) *C. elegans* nematode strain *glp-4;sek-1* exposed to *C. parapsilosis* strain ATCC# 22019 for 2 hours and then moved to microbe-free liquid media. A day later, there was a significant number of yeast cells within the nematode resulting in distention of the nematode intestine. The round structure (white arrows) is the pharyngeal grinder organ, which functions to disrupt ingested organisms. Black arrows with borders point to the intestinal lumen. (**b**) As the infection within the nematode progresses the nematode cells are essentially replaced by yeast cells and staining of nematodes with FUN-1 shows yeast cells to be metabolically active (*C. elegans glp-4;sek-1* nematode four days after infection with *C. parapsilosis* strain ATCC#22019).

**Figure S2. *In vitro* efficacy of CAPE, enoxacin and lapachol biofilm on the surfaces of wells of microtiter plates.** When evaluated using the XTT reduction assay CAPE and lapachol inhibited the formation of biofilm **(a)**, while all three compounds had an affect in pre-formed biofilm (**b**).

Supplemental Methods

**Effect of compounds on adherent cells and subsequent biofilm formation on the surfaces of wells of microtiter plates.** The effect of the compounds on adherent cells and subsequent biofilm formation was evaluated using the XTT reduction assay described by in [106]. The *C. albicans* strains DAY185 was inoculated in 2mL of RPMI 1640 and grown at 30ºC for 24h. Working concentrations of each compound were prepared in RPMI 1640. DMSO and dH_2_O were diluted with RPMI 1640 and used as controls.

**Effect of compounds on preformed biofilms on the surfaces of wells of microtiter plates.** The effect of compounds on preformed biofilms were evaluated as described in [106]. *C. albicans* biofilms were formed for 24h at 37°C on the surfaces of microtiter plates by using the protocol described above. After the biofilms were formed, the medium was aspirated, and non-adherent cells were removed by washing the biofilms three times in sterile PBS. Compounds were added into selected wells of the microtiter plates and plates were then incubated for 24 h at 37°C. The effect of each compound on preformed biofilms was then estimated by using the XTT reduction assay (above).

**Supplemental References**

1. Metzner J, Bekemeier H, Paintz M, Schneidewind E. (1979) [On the antimicrobial activity of propolis and propolis constituents]. Pharmazie. 34(2):97-102.

2. Elewski B, Ling MR, Phillips TJ (2006) Efficacy and safety of a new once-daily topical ketoconazole 2% gel in the treatment of seborrheic dermatitis: a phase III trial. J Drugs Dermatol 5: 646-650.

3. Bryckaert MC, Eldor A, Fontenay M, Gazit A, Osherov N, et al. (1992) Inhibition of platelet-derived growth factor-induced mitogenesis and tyrosine kinase activity in cultured bone marrow fibroblasts by tyrphostins. Exp Cell Res 199: 255-261.

4. Seidman LS, Skokos CK (2005) An evaluation of butoconazole nitrate 2% site release vaginal cream (Gynazole-1) compared to fluconazole 150 mg tablets (Diflucan) in the time to relief of symptoms in patients with vulvovaginal candidiasis. Infect Dis Obstet Gynecol 13: 197-206.

5. Sharif NA, Williams GW, DeSantis LM (1995) Affinities of muscarinic drugs for [3H]N-methylscopolamine (NMS) and [3H]oxotremorine (OXO) binding to a mixture of M1-M4 muscarinic receptors: use of NMS/OXO-M ratios to group compounds into potential agonist, partial agonist, and antagonist classes. Neurochem Res 20: 669-674.

6. Maass A, Kostenis E, Mohr K (1995) Potentiation by alcuronium of the antimuscarinic effect of N-methylscopolamine in guinea pig left atria. Eur J Pharmacol 272: 103-106.

7. Payette P, Gossard F, Whiteway M, Dennis M (1990) Expression and pharmacological characterization of the human M1 muscarinic receptor in *Saccharomyces cerevisiae*. FEBS Lett 266: 21-25.

8. Pereira EM, Machado Tde B, Leal IC, Jesus DM, Damaso CR, et al. (2006) *Tabebuia avellanedae* naphthoquinones: activity against methicillin-resistant staphylococcal strains, cytotoxic activity and in vivo dermal irritability analysis. Ann Clin Microbiol Antimicrob 5: 5.

9. Silva RS, Costa EM, Trindade UL, Teixeira DV, Pinto Mde C, et al. (2006) Synthesis of naphthofuranquinones with activity against *Trypanosoma cruzi*. Eur J Med Chem 41: 526-530.

10. Kitamura T, Nishimura S, Sasahara K, Yoshida M, Ando J, et al. (1999) Transplacental administration of diethylstilbestrol (DES) causes lesions in female reproductive organs of Donryu rats, including endometrial neoplasia. Cancer Lett 141: 219-228.

11. Tarry W, Fisher M, Shen S, Mawhinney M (2005) *Candida albicans*: the estrogen target for vaginal colonization. J Surg Res 129: 278-282.

12. Aubin MC, Carrier M, Shi YF, Tardif JC, Perrault LP (2006) Role of probucol on endothelial dysfunction of epicardial coronary arteries associated with left ventricular hypertrophy. J Cardiovasc Pharmacol 47: 702-710.

13. Jones BM, Kinghorn GR, Geary I (1982) In vitro susceptibility of *Gardnerella vaginalis* and *Bacteroides* organisms, associated with nonspecific vaginitis, to sulfonamide preparations. Antimicrob Agents Chemother 21: 870-872.

14. Layeeque R, Siegel E, Kass R, Henry-Tillman RS, Colvert M, et al. (2006) Prevention of nausea and vomiting following breast surgery. Am J Surg 191: 767-772.

15. Satoh Y, Sugiyama A, Takahara A, Ando K, Wang K, et al. (2005) The antipsychotic and antiemetic drug prochlorperazine delays the ventricular repolarization of the in situ canine heart. J Pharmacol Sci 97: 101-106.

16. Hendricks O, Molnar A, Butterworth TS, Butaye P, Kolmos HJ, et al. (2005) In vitro activity of phenothiazine derivatives in *Enterococcus faecalis* and *Enterococcus faecium*. Basic Clin Pharmacol Toxicol 96: 33-36.

17. Szefler SJ (1999) Pharmacodynamics and pharmacokinetics of budesonide: a new nebulized corticosteroid. J Allergy Clin Immunol 104: 175-183.

18. Gonzalo-Garijo MA, Rodriguez-Nevado I, de Argila D (2006) Patch tests for diagnosis of delayed hypersensitivity to cephalosporins. Allergol Immunopathol (Madr) 34: 39-41.

19. Schmidt AC (2005) Solid-state characterization of falicaine hydrochloride and isomorphic dyclonine hydrochloride. Part IV. Crystal polymorphism of local anaesthetic drugs. Eur J Pharm Sci 25: 407-416.

20. Giaever G, Flaherty P, Kumm J, Proctor M, Nislow C, et al. (2004) Chemogenomic profiling: identifying the functional interactions of small molecules in yeast. Proc Natl Acad Sci U S A 101: 793-798.

21. Pereda J, Noguera X, Boncompte E, Alguero M, Izquierdo I (2003) Efficacy of flutrimazole 1% powder in the treatment of tinea pedis. Mycoses 46: 126-131.

22. Fu H, Li W, Lao Y, Luo J, Lee NT, et al. (2006) Bis(7)-tacrine attenuates beta amyloid-induced neuronal apoptosis by regulating L-type calcium channels. J Neurochem.

23. Liu X, Zhu P, Freedman BD (2006) Multiple eicosanoid-activated nonselective cation channels regulate B-lymphocyte adhesion to integrin ligands. Am J Physiol Cell Physiol 290: C873-882.

24. Waterbury LD, Silliman D, Jolas T (2006) Comparison of cyclooxygenase inhibitory activity and ocular anti-inflammatory effects of ketorolac tromethamine and bromfenac sodium. Curr Med Res Opin 22: 1133-1140.

25. Fraser-Smith EB, Matthews TR (1987) Effect of ketorolac on *Candida albicans* ocular infection in rabbits. Arch Ophthalmol 105: 264-267.

26. Alem MA, Douglas LJ (2004) Effects of aspirin and other nonsteroidal anti-inflammatory drugs on biofilms and planktonic cells of *Candida albicans*. Antimicrob Agents Chemother 48: 41-47.

27. McCarthy RT, TanPiengco PE (1992) Multiple types of high-threshold calcium channels in rabbit sensory neurons: high-affinity block of neuronal L-type by nimodipine. J Neurosci 12: 2225-2234.

28. Berry EV, Toms NJ (2006) Pyruvate and oxaloacetate limit zinc-induced oxidative HT-22 neuronal cell injury. Neurotoxicology.

29. Parkinson JF (2006) Lipoxin and synthetic lipoxin analogs: an overview of anti-inflammatory functions and new concepts in immunomodulation. Inflamm Allergy Drug Targets 5: 91-106.

30. Ramstedt U, Ng J, Wigzell H, Serhan CN, Samuelsson B (1985) Action of novel eicosanoids lipoxin A and B on human natural killer cell cytotoxicity: effects on intracellular cAMP and target cell binding. J Immunol 135: 3434-3438.

31. Ramstedt U, Serhan CN, Nicolaou KC, Webber SE, Wigzell H, et al. (1987) Lipoxin A-induced inhibition of human natural killer cell cytotoxicity: studies on stereospecificity of inhibition and mode of action. J Immunol 138: 266-270.

32. Huang SM, Bisogno T, Petros TJ, Chang SY, Zavitsanos PA, et al. (2001) Identification of a new class of molecules, the arachidonyl amino acids, and characterization of one member that inhibits pain. J Biol Chem 276: 42639-42644.

33. Sipe JC, Arbour N, Gerber A, Beutler E (2005) Reduced endocannabinoid immune modulation by a common cannabinoid 2 (CB2) receptor gene polymorphism: possible risk for autoimmune disorders. J Leukoc Biol 78: 231-238.

34. Stratford AF, Zoutman DE, Davidson JS (2002) Effect of lidocaine and epinephrine on *Staphylococcus aureus* in a guinea pig model of surgical wound infection. Plast Reconstr Surg 110: 1275-1279.

35. Aydin ON, Eyigor M, Aydin N (2001) Antimicrobial activity of ropivacaine and other local anaesthetics. Eur J Anaesthesiol 18: 687-694.

36. Xiao CY, Hara A, Hashizume H, Tanaka K, Abiko Y (1999) Both D-cis- and L-cis-diltiazem attenuate hydrogen peroxide-induced derangements in rat hearts. Eur J Pharmacol 374: 387-398.

37. Osherov N, Levitzki A (1994) Epidermal-growth-factor-dependent activation of the src-family kinases. Eur J Biochem 225: 1047-1053.

38. Miyazaki Y, Shinomura Y, Tsutsui S, Yasunaga Y, Zushi S, et al. (1996) Oxidative stress increases gene expression of heparin-binding EGF-like growth factor and amphiregulin in cultured rat gastric epithelial cells. Biochem Biophys Res Commun 226: 542-546.

39. Gao YJ, Hirota S, Zhang DW, Janssen LJ, Lee RM (2003) Mechanisms of hydrogen-peroxide-induced biphasic response in rat mesenteric artery. Br J Pharmacol 138: 1085-1092.

40. Takase Y, Saeki T, Watanabe N, Adachi H, Souda S, et al. (1994) Cyclic GMP phosphodiesterase inhibitors. 2. Requirement of 6-substitution of quinazoline derivatives for potent and selective inhibitory activity. J Med Chem 37: 2106-2111.

41. Gallily R, Breuer A, Mechoulam R (2000) 2-Arachidonylglycerol, an endogenous cannabinoid, inhibits tumor necrosis factor-alpha production in murine macrophages, and in mice. Eur J Pharmacol 406: R5-7.

42. Lim EJ, Lee SH, Lee JG, Chin BR, Bae YS, et al. (2006) Activation of toll-like receptor-9 induces matrix metalloproteinase-9 expression through Akt and tumor necrosis factor-alpha signaling. FEBS Lett 580: 4533-4538.

43. Kettner C, Obermeyer G, Bertl A (2003) Inhibition of the yeast V-type ATPase by cytosolic ADP. FEBS Lett 535: 119-124.

44. Ibrahim-Granet O, Philippe B, Boleti H, Boisvieux-Ulrich E, Grenet D, et al. (2003) Phagocytosis and intracellular fate of *Aspergillus fumigatus* conidia in alveolar macrophages. Infect Immun 71: 891-903.

45. Medhora M, Harder D (1998) Functional role of epoxyeicosatrienoic acids and their production in astrocytes: approaches for gene transfer and therapy (review). Int J Mol Med 2: 661-669.

46. Beiner ME, Niv H, Haklai R, Elad-Sfadia G, Kloog Y, et al. (2006) Ras antagonist inhibits growth and chemosensitizes human epithelial ovarian cancer cells. Int J Gynecol Cancer 16 Suppl 1: 200-206.

47. Baker A, Northrop FD, Miedema H, Devine GR, Davies JM (2002) The non-steroidal anti-inflammatory drug niflumic acid inhibits *Candida albicans* growth. Mycopathologia 153: 25-28.

48. Tang KM, Jang EK, Haslam RJ (1994) Photoaffinity labelling of cyclic GMP-inhibited phosphodiesterase (PDE III) in human and rat platelets and rat tissues: effects of phosphodiesterase inhibitors. Eur J Pharmacol 268: 105-114.

49. Eskandari N, Wickramasinghe T, Peachell PT (2004) Effects of phosphodiesterase inhibitors on interleukin-4 and interleukin-13 generation from human basophils. Br J Pharmacol 142: 1265-1272.

50. Park WS, Chang YS, Lee M (2001) 3-Aminobenzamide, a poly (ADP-ribose) synthetase inhibitor, attenuates the acute inflammatory responses and brain injury in experimental *Escherichia coli* meningitis in the newborn piglet. Neurol Res 23: 410-416.

51. Azuara D, Sola A, Hotter G, Calatayud L, de Oca J (2005) Apoptosis inhibition plays a greater role than necrosis inhibition in decreasing bacterial translocation in experimental intestinal transplantation. Surgery 137: 85-91.

52. Ma YT, Gilbert BA, Rando RR (1993) Inhibitors of the isoprenylated protein endoprotease. Biochemistry 32: 2386-2393.

53. Chen Y, Ma YT, Rando RR (1996) Solubilization, partial purification, and affinity labeling of the membrane-bound isoprenylated protein endoprotease. Biochemistry 35: 3227-3237.

54. Rodriguez AB, Barriga C, de la Fuente M (1991) Stimulation of phagocytic processes and antibody-dependent cellular cytotoxicity of human neutrophils by cefmetazole. Microbiol Immunol 35: 545-556.

55. Vargas F, Mendez H, Tropper E, Velazquez M, Fraile G (2000) Studies on the in vitro phototoxicity of the antidiabetes drug glipizide. In Vitr Mol Toxicol 13: 17-24.

56. Dillon PF, Root-Bernstein RS, Lieder CM (2004) Antioxidant-independent ascorbate enhancement of catecholamine-induced contractions of vascular smooth muscle. Am J Physiol Heart Circ Physiol 286: H2353-2360.

57. Sordillo LM, Weaver JA, Cao YZ, Corl C, Sylte MJ, et al. (2005) Enhanced 15-HPETE production during oxidant stress induces apoptosis of endothelial cells. Prostaglandins Other Lipid Mediat 76: 19-34.

58. Yilmaz O, Celik S (2003) A combination of alpha-tocopherol, vitamin C and N-acetyl cysteine increases unsaturated fatty acid levels in hydrogen peroxide-induced *Candida tropicalis* (ATCC 13803). Cell Biochem Funct 21: 269-274.

59. Costamagna L, Rosi I, Garuccio I, Arrigoni O (1986) Ascorbic acid specific utilization by some yeasts. Can J Microbiol 32: 756-758.

60. Anderson R (1981) Ascorbate-mediated stimulation of neutrophil motility and lymphocyte transformation by inhibition of the peroxidase/H2O2/halide system in vitro and in vivo. Am J Clin Nutr 34: 1906-1911.

61. Cano-Abad MF, Villarroya M, Garcia AG, Gabilan NH, Lopez MG (2001) Calcium entry through L-type calcium channels causes mitochondrial disruption and chromaffin cell death. J Biol Chem 276: 39695-39704.

62. Luo T, Fredericksen BL, Hasumi K, Endo A, Garcia JV (2001) Human immunodeficiency virus type 1 Nef-induced CD4 cell surface downregulation is inhibited by ikarugamycin. J Virol 75: 2488-2492.

63. Doggrell S, Hoey A, Brown L (1994) Ion channel modulators as potential positive inotropic compound for treatment of heart failure. Clin Exp Pharmacol Physiol 21: 833-843.

64. Theodoropoulos PA, Gravanis A, Tsapara A, Margioris AN, Papadogiorgaki E, et al. (1994) Cytochalasin B may shorten actin filaments by a mechanism independent of barbed end capping. Biochem Pharmacol 47: 1875-1881.

65. Hamano H, Nabekura J, Nishikawa M, Ogawa T (1996) Docosahexaenoic acid reduces GABA response in substantia nigra neuron of rat. J Neurophysiol 75: 1264-1270.

66. Hamam F, Shahidi F (2006) Synthesis of structured lipids containing medium-chain and omega-3 fatty acids. J Agric Food Chem 54: 4390-4396.

67. Vestergaard C, Johansen C, Otkjaer K, Deleuran M, Iversen L (2005) Tumor necrosis factor-alpha-induced CTACK/CCL27 (cutaneous T-cell-attracting chemokine) production in keratinocytes is controlled by nuclear factor kappaB. Cytokine 29: 49-55.

68. Conconi M, Petropoulos I, Emod I, Turlin E, Biville F, et al. (1998) Protection from oxidative inactivation of the 20S proteasome by heat-shock protein 90. Biochem J 333 (Pt 2): 407-415.

69. Chen JC, Chen JQ, Xie QM, Zhu YL (2004) Selective inhibition of purified human phosphodiesterase 4A expressed in yeast cell GL62 by ciclamilast, piclamilast, and rolipram. Acta Pharmacol Sin 25: 1171-1175.

70. Alexander RP, Warrellow GJ, Eaton MA, Boyd EC, Head JC, et al. (2002) CDP840. A prototype of a novel class of orally active anti-inflammatory phosphodiesterase 4 inhibitors. Bioorg Med Chem Lett 12: 1451-1456.

71. Sun Y, Li L, Lau F, Beavo JA, Clark EA (2000) Infection of CD4+ memory T cells by HIV-1 requires expression of phosphodiesterase 4. J Immunol 165: 1755-1761.

72. Meydan N, Grunberger T, Dadi H, Shahar M, Arpaia E, et al. (1996) Inhibition of acute lymphoblastic leukaemia by a Jak-2 inhibitor. Nature 379: 645-648.

73. Dadi H, Ke S, Roifman CM (1994) Activation of phosphatidylinositol-3 kinase by ligation of the interleukin-7 receptor is dependent on protein tyrosine kinase activity. Blood 84: 1579-1586.

74. Sharfe N, Dadi HK, Roifman CM (1995) JAK3 protein tyrosine kinase mediates interleukin-7-induced activation of phosphatidylinositol-3' kinase. Blood 86: 2077-2085.

75. Anastassiades T, Chopra R, Law C, Wong E (1998) In vitro suppression of transforming growth factor-beta induced stimulation of glycosaminoglycan synthesis by acetylsalicylic acid and its reversal by misoprostol. J Rheumatol 25: 1962-1967.

76. Hayes MM, Lane BR, King SR, Markovitz DM, Coffey MJ (2002) Prostaglandin E(2) inhibits replication of HIV-1 in macrophages through activation of protein kinase A. Cell Immunol 215: 61-71.

77. Zhang Z, ElSohly HN, Jacob MR, Pasco DS, Walker LA, et al. (2002) Natural products inhibiting *Candida albicans* secreted aspartic proteases from *Tovomita krukovii*. Planta Med 68: 49-54.

78. Shin EH, Lee HY, Bae YS (2006) Leukotriene B4 stimulates human monocyte-derived dendritic cell chemotaxis. Biochem Biophys Res Commun 348: 606-611.

79. Bibel DJ, Aly R, Shah S, Shinefield HR (1993) Sphingosines: antimicrobial barriers of the skin. Acta Derm Venereol 73: 407-411.

80. Gueguen G, Gaige B, Grevy JM, Rogalle P, Bellan J, et al. (1999) Structure-activity analysis of the effects of lysophosphatidic acid on platelet aggregation. Biochemistry 38: 8440-8450.

81. Beekwilder JP, van Kempen GT, van den Berg RJ, Ypey DL (2006) The local anesthetic butamben inhibits and accelerates low-voltage activated T-type currents in small sensory neurons. Anesth Analg 102: 141-145.

82. Gonzalez-Aragon D, Buron MI, Lopez-Lluch G, Herman MD, Gomez-Diaz C, et al. (2005) Coenzyme Q and the regulation of intracellular steady-state levels of superoxide in HL-60 cells. Biofactors 25: 31-41.

83. Feuerstein G, Boonyaviroj P, Gutman Y (1979) The effect of saline loading on blood pressure and catecholamine secretion in the rat and the cat. Eur J Pharmacol 54: 373-382.

84. Currie GP, Paterson E, Keenan F, Nath S, Watt SJ (2005) An unexpected response to intravenous hydrocortisone succinate in an asthmatic patient. Br J Clin Pharmacol 60: 342.

85. Jauch R, Bozler G, Hammer R, Koss FW, Karlsson M, et al. (1978) [Ambroxol, studies of biotransformation in man and determination in biological samples (author's transl)]. Arzneimittelforschung 28: 904-911.

86. Capsoni F, Ongari AM, Minonzio F, Lazzarin A, Zavattini G (1985) Effect of ambroxol on human phagocytic cell function. Boll Ist Sieroter Milan 64: 236-239.

87. Su X, Wang L, Song Y, Bai C (2004) Inhibition of inflammatory responses by ambroxol, a mucolytic agent, in a murine model of acute lung injury induced by lipopolysaccharide. Intensive Care Med 30: 133-140.

88. Kronig B, Pittrow DB, Kirch W, Welzel D, Weidinger G (1997) Different concepts in first-line treatment of essential hypertension. Comparison of a low-dose reserpine-thiazide combination with nitrendipine monotherapy. German Reserpine in Hypertension Study Group. Hypertension 29: 651-658.

89. Selvaag E (1996) Inhibition of thiazide photohemolysis in vitro by antioxidants and a nitrogen atmosphere. Photodermatol Photoimmunol Photomed 12: 211-215.

90. Nishimori I, Minakuchi T, Morimoto K, Sano S, Onishi S, et al. (2006) Carbonic anhydrase inhibitors: DNA cloning and inhibition studies of the alpha-carbonic anhydrase from *Helicobacter pylori*, a new target for developing sulfonamide and sulfamate gastric drugs. J Med Chem 49: 2117-2126.

91. Cohen S, Samadikuchaksaraei A, Polak JM, Bishop AE (2006) Antibiotics reduce the growth rate and differentiation of embryonic stem cell cultures. Tissue Eng 12: 2025-2030.

92. Moulin P, Andre M, Alawi H, dos Santos LC, Khalid AK, et al. (2006) Efficacy of benfluorex in combination with sulfonylurea in type 2 diabetic patients: an 18-week, randomized, double-blind study. Diabetes Care 29: 515-520.

93. Larschan E, Winston F (2005) The *Saccharomyces cerevisiae* Srb8-Srb11 complex functions with the SAGA complex during Gal4-activated transcription. Mol Cell Biol 25: 114-123.

94. Petrou MA, Rogers TR (1988) In-vitro activity of antifungal agents in combination with four quinolones. Drugs Exp Clin Res 14: 9-18.

95. Siminiak T, Wysocki H, Veit A, Maurer HR (1991) The effect of selected antiarrhythmic drugs on neutrophil free oxygen radicals production measured by chemiluminescence. Basic Res Cardiol 86: 355-362.

96. Raab WP, Gmeiner BM (1975) Inhibition of glucose-6-phosphate dehydrogenase activity by betamethasone and three of its esters with dermatological importance. Arch Dermatol Res 253: 113-118.

97. Maraki S, Mouzas IA, Kontoyiannis DP, Chatzinikolaou I, Tselentis Y, et al. (2001) Prospective evaluation of the impact of amoxicillin, clarithromycin and their combination on human gastrointestinal colonization by *Candida* species. Chemotherapy 47: 215-218.

98. Cascio C, Guarneri R, Russo D, De Leo G, Guarneri M, et al. (2002) A caspase-3-dependent pathway is predominantly activated by the excitotoxin pregnenolone sulfate and requires early and late cytochrome c release and cell-specific caspase-2 activation in the retinal cell death. J Neurochem 83: 1358-1371.

99. Rezanka T, Dolezalova L, Vyhnalek O, Novotny C (1985) Effect of clomiphene on the content of sterols and fatty acids in Saccharomyces cerevisiae. Folia Microbiol (Praha) 30: 501-505.

100. Lyte M, Freestone PP, Neal CP, Olson BA, Haigh RD, et al. (2003) Stimulation of *Staphylococcus epidermidis* growth and biofilm formation by catecholamine inotropes. Lancet 361: 130-135.

101. Franco R, Cidlowski JA (2006) SLCO/OATP-like transport of glutathione in FasL-induced apoptosis: Glutathione efflux is coupled to an organic anion exchange and is necessary for the progression of the execution phase of apoptosis. J Biol Chem.

102. Sabie FT, Gadd GM (1992) Effect of nucleosides and nucleotides and the relationship between cellular adenosine 3':5'-cyclic monophosphate (cyclic AMP) and germ tube formation in *Candida albicans*. Mycopathologia 119: 147-156.

103. Srikrishnan T, Parthasarathy R (1985) Intercalation of water molecules between nucleic acid bases in the crystal structures of 6-azathymine hemihydrate and 5-amino-2-thiocytosine dihydrochloride dihydrate. Arch Biochem Biophys 239: 38-45.

104. Spassov A, Golovinsky E, Spassovska N, Maneva L (1972) Effect of thio- and hydrazino-derivatives of uracil, 6-azauracil and 6-azathymine on the growth of some microorganisms in vitro. Z Naturforsch [B] 27: 818-821.

105. Lhotta K, Gruber J, Sgonc R, Fend F, Konig P (1998) Apoptosis of tubular epithelial cells in familial juvenile gouty nephropathy. Nephron 79: 340-344.

106. Ramage G, Saville SP, Wickes BL, Lopez-Ribot JL (2002) Inhibition of *Candida albicans* biofilm formation by farnesol, a quorum-sensing molecule. Appl Environ Microbiol 68: 5459-5463.
